# The role of FET-PET in patient selection and response assessment for reirradiation in recurrent glioblastoma

**DOI:** 10.3389/fonc.2025.1604448

**Published:** 2025-08-07

**Authors:** Izabela Zarębska, Maciej Blok, Michał Marjański, Maciej Harat

**Affiliations:** ^1^ Department of Neurooncology and Radiosurgery, Franciszek Lukaszczyk Oncology Center, Bydgoszcz, Poland; ^2^ Department of Radiotherapy, Franciszek Lukaszczyk Oncology Center, Bydgoszcz, Poland; ^3^ Department of Clinical Medicine, Jan and Jędrzej Śniadecki University of Science and Technology in Bydgoszcz, Bydgoszcz, Poland; ^4^ Department of Clinical Oncology, Franciszek Lukaszczyk Oncology Center, Bydgoszcz, Poland

**Keywords:** glioblastoma, amino acid PET, post-treatment changes, tumor progression, response assessment

## Abstract

Radiation treatment with modern techniques is frequently used in the complex interdisciplinary management of glioblastoma, both in primary and recurrent setting. However, standard imaging limits the ability to precisely distinguish treatment-related changes from tumor progression and to accurately assess the outcomes of radiotherapy. Given the challenges, there is growing need for advanced imaging modalities that can enhance diagnostic precision and guide therapeutic decision. One such modality is FET-PET, whose role in glioblastoma radiotherapy is increasingly recognized - from target definition to response assessment and differentiation between progression and post-irradiation changes. Recently, PET RANO criteria have been published, providing an optimal strategy for evaluating treatment response using amino-acid PET. Earlier, contributions from the PET/RANO group also supported the integration of PET imaging in radiotherapy planning and monitoring of glioma patients. Increasing evidence highlights the advantages of amino-acid PET over standard RANO MRI in predicting overall survival. However, its value in case of patient selection and monitoring of reirradiation is less clear. In this narrative review, we aimed to summarize the published data on FET-PET in patients after initial treatment from the perspective of radiation oncologist. We focused on the role of FET-PET in accurate diagnosis of recurrence and in treatment response after reirradiation.

## 1 Introduction

Glioblastoma is the most common malignant brain tumor in adults. Standard treatment includes maximal safe resection followed by radiotherapy with concomitant and adjuvant temozolomide. Despite advances in oncological therapies, glioblastoma remains associated with tremendously poor prognosis – median overall survival (OS) ranges from 14 to 18 months and median progression-free survival (PFS) is approximately 10 to 12 months (1–4).

Early diagnosis of treatment failure to initiate salvage therapy remains controversial due to limited therapeutic options and the low accuracy of MRI in reliably determining the true extent of disease. Moreover, conventional MRI sequences often fail to distinguish post-treatment changes - such as radionecrosis and pseudoprogression - and actual tumor progression, which may lead to inappropriate treatment decisions.

Posttreatment changes on conventional MRI vary depending on the type of previous treatment. Radiotherapy-induced changes are typically located within areas that received the highest radiation dose – the most often near resection cavity or tumor bed. These post-radiotherapy changes may include Swiss-like and bubble-like patterns, oedema and mass effect. On T2-weighted images, radiation-induced alternations are characterized by low signal intensity with a centrally increased signal corresponding to necrosis. Additional findings may include enhancement of the white matter and cortex (5). Changes after treatment with bevacizumab with or without lomustine are commonly manifested by the reduction in contrast enhancement and oedema (6, 7). Response assessment criteria based on MRI findings of adult gliomas have been described in RANO 2.0 (8).

In recent years, PET imaging has gained prominence for evaluating treatment response (9–11). The most commonly used PET tracer in oncology is ^18^F-fluorodeoxyglucose ([^18^F] FGD). However, its diagnostic utility in brain tumors is limited due to high physiological glucose uptake in normal brain tissue (12, 13). Gliomas are characterized by the overexpression of L-amino acid transporters compared to normal brain cells (14). It enables the use of amino acid tracers such as ^11^ C-methionine ([^11^C] MET), ^18^ F-dihydroxyphenylalanine ([^18^ F] F-DOPA) and ^18^ F-fluoroethyl-L-tyrosine ([^18^ F] FET) in neuro-oncology.

These tracers have the unique ability to cross blood-brain barrier (BBB) and to visualize tumor extent beyond the areas of contrast enhancement seen on MRI or even beyond FLAIR (15, 16). Compared to conventional MRI, PET offers higher sensitivity and specificity for detecting neoplastic infiltration, superior assessment of metabolic response to treatment, greater accuracy in differentiating tumor progression from radiation-induced changes and response to treatment (9, 17, 18). The recently published ESTRO/EANO recommendations on reirradiation of glioblastoma endorse the addition of amino acid PET to assess recurrence after primary treatment (19). FET-PET demonstrates excellent sensitivity of 0.82 and specificity of 0.76 in the diagnosis of primary brain tumors (20). FET is characterized by high *in vivo* stability and easy synthesis (21). Its concentration is significantly increased in neoplastic lesions compared to healthy or infected tissues (22, 23). The high intracellular accumulation of FET is attributed to elevated LAT1–3 expression on glioma cells as well as its molecular weight of approximately 220 g/mol (24, 25). Another advantage of FET is its half-life of 110min, which makes is suitable for clinical use (26). FET uptake and kinetics in glioma are generally stable, but tumor-to-background ratio (TBR) might slightly decrease due to dexamethasone administration (25). General recommendations for the use of PET in glioma patients have been summarized by the PET/RANO working group and in EANM/EANO/RANO/SNMMI guidelines (27, 28). More recently, PET RANO 1.0 criteria have been introduced to standardize treatment response assessment based on PET imaging (29). In response to growing interest in amino acid PET tracers in neuro-oncology, PET RANO BM 1.0 criteria were also published to evaluate the metabolic response of brain metastases (30).

For radiation oncologists, accurate diagnosis of recurrence is particularly crucial and requires advanced imaging. Recently published recommendations on the use of FET-PET focus only on treatment assessment after first-line therapy and do not address other challenges such as post-treatment changes, selection criteria for retreatment and response assessment after re-irradiation. Therefore, we aimed to summarize the available evidence on the use of FET-PET in clinical scenarios following initial treatment: in patients with residual tumor, patients with enlarging lesions, patients with new, small areas of contrast enhancement and after retreatment.

## 2 Methods

**Figure 1:**
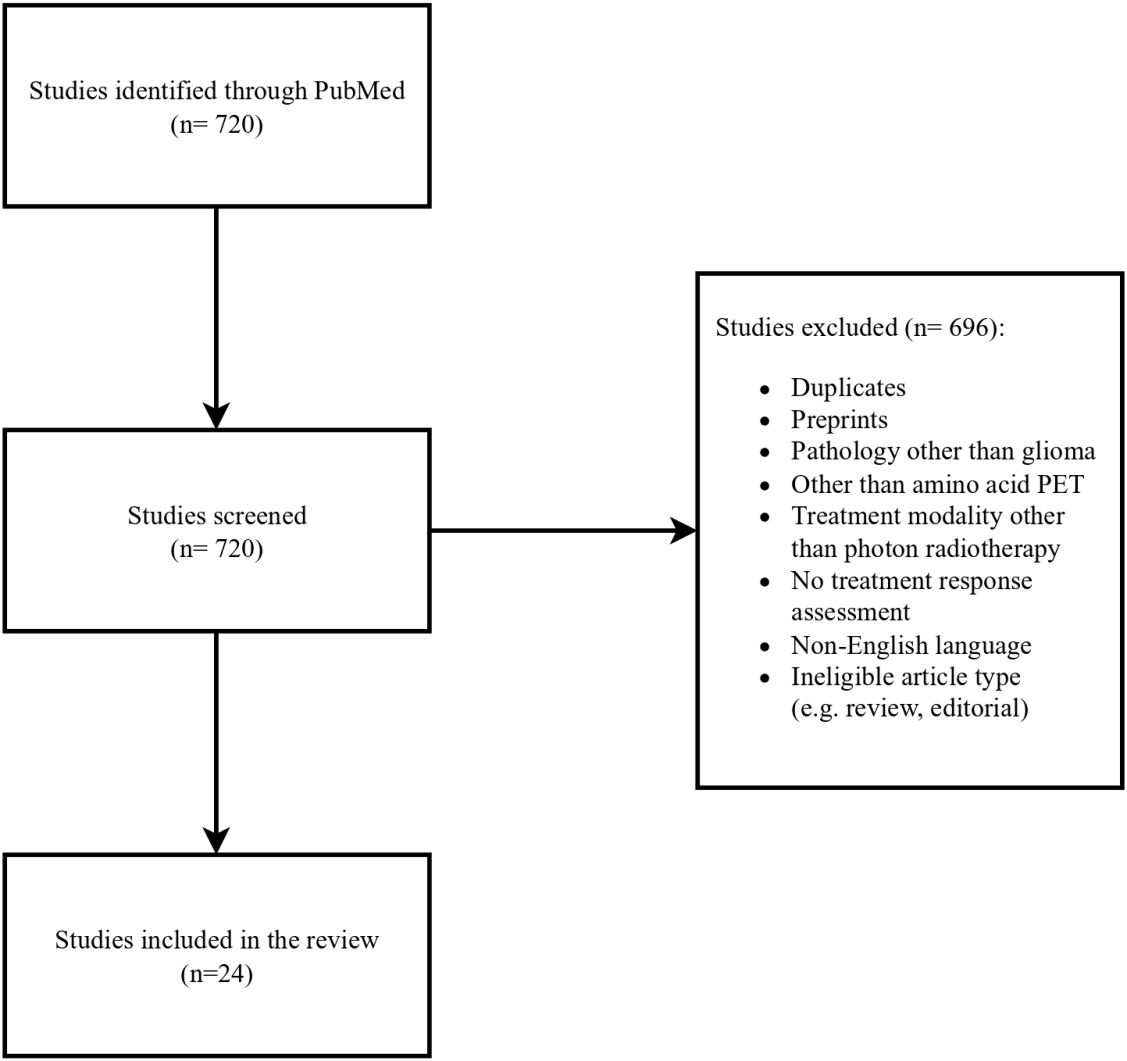
Flow chart of the literature selection process.

The authors conducted a literature search of published journal articles written in English between January and May 2025 in PubMed. Keywords such as:” glioblastoma”, “primary brain tumors”, “FET”, “PET”, “recurrent glioblastoma”, “treatment-related changes”, “PET/RANO” and” RANO” were used. Retrospective studies, prospective studies and systematic reviews were analyzed. A workflow of the literature selection process is illustrated in **Figure 1**.

## 3 FET-PET in patients with residual tumor after initial treatment

The optimal management and prognosis of patients with residual, stable tumor on MRI after radiochemotherapy remain unclear. The association between prognostic factors such as OS and PFS and both static (e.g. TBR_mean/max_ – tumor-to-brain ratio; MTV- metabolic tumor volume; BTV - biological tumor volume) and dynamic (TTP – time to peak; TAC – time-activity curve) FET-PET parameters after glioblastoma treatment has been evaluated in five prospective studies.

In a study by Galldiks et al., 25 patients with glioblastoma were evaluated with MRI and FET-PET at different timepoints following standard treatment – after surgery, 7–10 days after radiochemotherapy and 6–8 weeks later. The results showed that a ≥ 10% decrease in both TBR_max_ and TBR_mean_ on FET-PET done 7–10 days after radiochemotherapy was a favorable prognostic factor for PFS (TBR_max_ 9.3 vs. 4.7 months; p= 0.002; TBR_mean_ 10.3 vs. 5.1 months p <0.001) and OS (TBR_max_ 15.4 vs. 8.5 months; p = 0.001; TBR_mean_ 16.1 vs. 9.3 months, p<0.001). FET-PET performed 6–8 weeks later had a lower predictive value for TBR_max/mean_ but a reduction in T_VOL1.6_ (tumor volume with TBR > 1.6) was associated with improved PFS (9.3 vs. 5.1 months; p = 0.002). MRI-based tumor volume changes were not associated with survival (31).

Another study by Piroth et al. analyzed static and dynamic FET-PET parameters in 25 glioblastoma patients at the same timepoints. They found that a ≥ 10% decrease in TBR_max_ on FET-PET after radiochemotherapy (7–10 days) was associated with longer median PFS (9.3 vs 4.7 months; p =0.002) and OS (18.0 vs 8.5 months; p <0.01). Similarly, a ≥5% reduction in TBR_mean_ (cutoff 5%) was associated with longer PFS (10.3 vs 5.1 months) and OS (22.8 vs 9.3 months), with p < 0.001 for both. However, changes in TTP and the slope of the TAC were not associated with survival outcomes (32).

A study by Ceccon et al. included 41 glioma patients (90% diagnosed with WHO grade 4 glioblastoma) who received surgery, radiochemotherapy and adjuvant temozolomide. FET-PET performed after two cycles of adjuvant temozolomide showed that a reduction in TBR_max_ and MTV compared to baseline was associated with longer OS (24 vs. 12 months; p =0.032, and 29 vs. 12 months; p = 0.005) and PFS (both 11 vs. 8 months; p = 0.031 and 0.007, respectively). No significant correlations were observed between MRI findings and OS or PFS (33).

A prospective study by Suchorska et al. assessed both static and dynamic FET-PET in 79 patients with newly diagnosed glioblastoma treated with surgery, radiochemotherapy with temozolomide and adjuvant temozolomide. FET-PET imaging was performed before and after surgery, 4–6 weeks after radiochemotherapy and after four cycles of adjuvant temozolomide. A smaller BTV (below 9.5cm^3^, sensitivity 64%, specificity 70%) before radiochemotherapy was identified as a prognostic factor. Median OS and PFS for BTV below 9.5 cm^3^ were 17.5 and 8.8 months (p<0,002), respectively, compared to 10.7 and 3.9. months for BTV above 9.5cm^3^ (p <0.08). Additionally, patients with initially increased TACs had longer OS (29.7 vs 12.5 months; p <0.02, HR 2.1) and longer PFS (11.9 vs 5.8 months; p < 0.05, HR 1.8) (34).

Finally, a prospective study by Harat et al. evaluated the use of a simultaneous integrated boost planned on FET-PET in the postoperative treatment of 17 patients with newly diagnosed glioblstoma. The study demonstrated that FET-PET-based treatment response assessment enables accurate evaluation of biological response. In 81% of analyzed cases, there was discordance between PET and MRI-based RANO criteria for response assessment (35). In summary, FET-PET may be considered as a reliable tool in early treatment response evaluation and survival prediction. Abovementioned studies have been summarized in **Table 1**.

**Table 1:** Overview of studies analyzing prognostic value of FET-PET.

Study	N of pts	Time of PET after irradiation	Evaluated parameters	Dynamic vs static acquisition	Prognostic value
Galldiks et al. (31)	25	7–10 days and 6–8 weeks after RTH	TBR_mean_, TBR_max_, Tvol	Static	A decrease of TBR_max_ and TBR_mean_ in early PET predicted longer PFS and OS. In FET-PET done 6-8 weeks later Tvol decrease was related to longer PFS.
Piroth et al. (32)	25	7–10 days and 6–8 weeks after RTH	TBR_max_, TBR_mean_, TTP, TAC	Static and dynamic	Decrease of TBR_mean_ and TBR_max_ after RTH – longer PFS and OS. No significant correlation of dynamic parameters and survival.
Ceccon et al. (33)	41	7 days before adjuvant TMZ and after 2 cycle of adjuvant TMZ	TBR_max_, TBR_mean_, MTV	Static	Reductions of MTV and TBR_max_ predicted longer OS and PFS.
Suchorska et al. (34)	79	4–6 weeks after RTH and after 3 cycles of TMZ	BTV, TAC	Static and dynamic	Longer OS and PFS in patients with smaller pretreatment BTV. Initially increased TAC associated with longer PFS.
Harat et al. (35)	11	3–8 months after RTH	MTV	Dual time static aquisition	Relative changes in PET volume and PET volume at time of response assessment were associated with OS.

RTH, radiotherapy; TMZ, temozolomide; TBR, tumor to background ratio; Tvol, tumor volume; BTV, biological tumor volume; TAC, time activity curve; TTP, time to peak; MTV, metabolic tumor volume; OS, overall survival; PFS, progression free survival.

## 4 FET-PET in patients with progressive enlarging of a mass lesion

After radiochemotherapy a progression of contrast enhancement on MRI might represent both tumor progression and radionecrosis.

Radionecrosis is a local tissue response to radiotherapy and typically develops within regions exposed to the highest dose of radiation. The risk of radionecrosis increases with higher total dose, larger fraction size and concurrent chemotherapy. Treatment-related necrosis can occur up to 30% of patients. In contrast to pseudoprogression, radionecrosis generally develops more than three months after treatment, although it can also manifest years or even decades later. On MRI images, radionecrosis can be characterized by contrast enhancement, which may be accompanied by clinical symptoms (34–36). Symptomatic radionecrosis can be managed with steroids, bevacizumab and surgery (36, 37). While biopsy can distinguish necrosis from recurrence, it carries surgical risks and might be limited due to sampling errors that do not represent the full pathological profile of the tumor (38–40). FET-PET might help to distinguish post-treatment necrotic tissue from tumor progression. However, most studies are retrospective and include relatively small patient cohorts with heterogenous histopathology.

A systematic review analyzing three studies on the use of FET-PET for the differential diagnosis of radionecrosis and glioma recurrence demonstrated higher diagnostic accuracy compared to FDG-PET (specificity 78–95% versus 70–88% for FDG and sensitivity 82–91% for FET 70–84% for FDG) (41).

The diagnostic accuracy, sensitivity and specificity of MRI were reported as 93.75%, 96%, and 85.7%, respectively. When both MR and FET-PET were evaluated, these values increased to 96.87%, 100%, and 85.7%, respectively. TBR_max_ cut-off (threshold) of 2.09 provided sensitivity of 100% and specificity of 72% for distinguishing recurrence from radionecrosis. A TBR_mean_ cut-off of 1.517 yielded a slightly lower specificity but higher sensitivity – 89% and 86% (42).

Another study showed that similar TBR_max_ cut-off 2.07 (measured 30–40 min. postinjection) resulted in sensitivity and specificity of 80% and 84.6%, respectively (43).

In contrast, a study by Vidmar reported higher value TBR_max_ cut-off - 3.03 (sensitivity and specificity of 77% and 82%) (44). The discrepancies in TBR tresholds might be attributed to small, heterogenous study populations, the lack of histopathological examination, and differences in PET protocols and interpretation.

A retrospective study by Bashir et al. involving 146 glioblastoma patients showed that FET-PET parameters measured at least 6 months post-treatment were significantly higher in recurrent glioblastoma than in post-treatment changes (TBR_max_ 3.2 vs 1.6; TBR_mean_ 2.0 vs 1.6; and BTV 14.8 cm^3^ vs. 0.01 cm^3^; p < 0.0001). Optimal thresholds were identified as 2.0 for TBR_max_ and 1.8 for TBR_mean_ and 0.55 cm^3^ for BTV to accurately differentiate tumor progression from treatment-related changes. Increasing TBR_max_ (HR 1.328, 95% CI: 1.116–1.582; p = 0.001) and BTV during follow-up (HR 1.303, 95% CI: 1.179–1.439; p < 0.0001) were associated with shorter OS (45).

Another retrospective study by Werner et al. evaluated 48 patients with high grade glioma and suspicious MRI findings. FET-PET demonstrated significantly superior diagnostic performance (threshold for both TBR_max_ and TBR_mean_: 1.95; accuracy: 83%; p < 0.001) compared to apparent diffusion coefficient (ADC) values (threshold:1.09 × 10^−3^ mm^2^/s; accuracy, 69%; p = 0.13). The optimal TTP cut-off of 32.5 min was optimal for the differentiation (accuracy 72%; sensitivity 80%; specificity 69%; p< 0.01) and for slope - 0.32 SUV/h (change of SUV per hour) with an accuracy of 74%, sensitivity of 70% and specificity of 75% (p= 0.02). Combining static FET-PET parameters with ADC increased diagnostic accuracy to 89%, while combining both static and dynamic FET-PET parameters yielded the highest accuracy (93%). TBR_max_ and TBR_mean_ values <1.95 were associated with longer OS (p = 0.01) (46).

Celli et al. retrospectively analyzed 45 glioma patients (26 with WHO grade 4 glioblastoma) and found similar sensitivity (86.2%) and specificity (81.31%) for FET-PET in the differential diagnosis performed at least 12 weeks after radiotherapy. The optimal cut-offs for recurrence were consistent with previous findings (TBR_max_ ≥ 2.1, SUV_max_ ≥ 3.5, and TTP ≤ 29 min). However, no FET-PET parameters significantly impacted overall survival (47).

Maurer et al. conducted a retrospective study of 147 glioma patients (67 with WHO grade 4 glioblastoma). The diagnostic performance of TBR_max_ and TBR_mean_ thresholds of 1.95 was slightly lower (sensitivity 70%; specificity 71%; accuracy 70%; for TBRmax and sensitivity 56%; specificity 79%; accuracy 62%; for TBRmean). The optimal cut-off for slope was lower – 0.2SUV/h (sensitivity 54%; specificity 86%; accuracy 63%);. Nonetheless, TBR_max_ > 1.95 combined with a slope < 0.2 SUV/h achieved a sensitivity of 86%, a specificity of 67%, and an accuracy of 81% in detecting progression (48).

In summary, static (TBR_max/mean_ < 2) and dynamic (TTP >30min) FET-PET parameters can differentiate necrosis from tumor progression. Diagnostic accuracy can be further improved by combining FET-PET with MRI-derived parameters, such as ADC. However, these improvements do not appear to translate into significantly longer OS.

## 5 FET-PET in patients with new, small areas of contrast enhancement

Management of asymptomatic patients with new, small, multicentric lesions remains challenging, as such MRI findings might indicate either tumor progression or pseudoprogression.

The pathogenesis of pseudoprogression remains unclear, and no universally accepted definition currently exists. However, it is often described as at least 25% increase in tumor size according to Macdonald criteria, followed by either partial response or stable disease lasting for at least six months after radiochemotherapy (49).

Pseudoprogression is more commonly observed in patients with MGMT promoter methylation and in those treated with radiochemotherapy including temozolomide, compared to radiotherapy alone (50, 51).

Similar to necrosis, pseudoprogression can mimic tumor progression on MRI. Histopathological examination may reveal residual, stable tumor tissue within areas of pseudoprogession and necrosis, potentially leading to a wrong diagnosis of progression (39, 52–55).

Studies of FET-PET in patients with suspected MRI findings showed promising results.

A retrospective study by Galldiks et al. included 22 glioblastoma patients with suspicious MRI findings within the first 12 weeks after radiochemotherapy. In patients with pseudoprogression, FET uptake was significantly lower than in those with true progression (TBR_max_ 1.9 ± 0.4 vs. 2.8 ± 0.5, TBR_mean_ 1.8 ± 0.2 vs. 2.3 ± 0.3; p<0.001). TTP was shorter in progression than in pseudoprogression (mean TTP 26 ± 10 vs. 35 ± 9 min, *P* = 0.05). TACs type II (uptake peaking at a mid-point; >20–40 min) or III (early uptake peak ≤20 min followed by a constant decline) were more frequently observed in progression (p=0.04). The optimal TBR_max_ cut-off for identifying pseudoprogression was 2.3 (sensitivity 100%, specificity 91%, accuracy 96%, p<0.001) (56).

Another study by Kebir et al. reported comparable results in 26 glioblastoma patients with MRI changes observed at least 12 weeks post-radiotherapy. TBR_max_ and TBR_mean_ were significantly higher in patients with progression compared to those with late pseudoprogression (TBR_max_ 2.4 ± 0.1 vs. 1.5± 0.2, P =0.003; TBR_mean_ 2.1 ± 0.1 vs. 1.5 ± 0.2, p =0.012). The optimal cut-off for both parameters was 1.9 (TBR_max_: sensitivity 84%, specificity 86%, accuracy 85%, p =0.015; TBR_mean_: sensitivity 74%, specificity 86%, accuracy 77%, p= 0.023). The authors suggested to diagnosing late progression when TBR_max_ exceeds 2.4 and late pseudoprogression when it is below 1.0; values between 1.0 and 2.4 should be interpreted with caution. TTP was significantly shorter in progression (mean TTP 25 ± 2 vs. 40 ± 2 min, p< 0.001). TACs type II or III were more frequently observed in progression, with a sensitivity of 84%, specificity of 100%, and an accuracy of 89% (p< 0.001) (57).

A retrospective study by Werner et al. analyzed 23 glioblastoma patients with progressive MRI findings after chemoradiation with lomustine and temozolomide. FET-PET were consistent with previous studies and significantly contributed to diagnosing pseudoprogression. Lower TBR_mean/max_ values were observed in pseudoprogression compared to progression (TBR_mean_ 1.9 ± 0.2 vs. 2.1 ± 0.2; *P* = 0.023 and TBR_max_ 2.8 ± 0.6 vs. 3.2 ± 0.5; *P* = 0.045), while TTP was higher in pseudoprogression (36.6 ± 8.3 vs. 24.8 ± 9.4 minutes; *p* = 0.005). The optimal cut-off values were: TBR_mean_ 1.95 (sensitivity 82%; specificity 92%; accuracy 87%; *P* = 0.029), TBR_max_ 2.85 (sensitivity 64%; specificity 92%; accuracy 78%; *P* = 0.046) and TTP 35 minutes (sensitivity, 64%; specificity, 83%; accuracy 74 P = 0.010). Combining TBR_mean_ with TTP improved the specificity and positive predictive value to 100% for detecting pseudoprogression (sensitivity 55%; accuracy 78%; *P* = 0.005) (58).

In contrast, a study by Mihovilovic et al. reported higher FET-PET parameters in 36 glioblastoma patients with suspected recurrence. The optimal tresholds for differentiating progression from late pseudoprogression were: TBR_max_ of 3.52 (sensitivity 89%, specificity 75%, area under the curve (AUC) 0.87 ± 0.07; p=0.0020) and TBR_mean_ of 2.98 (sensitivity 82%, specificity 87.5%, AUC 0.84 ± 0.08, p=0.004) (59).

In conclusion, current evidence supports the utility of FET-PET in distinguishing progression from pseudoprogression. Static and dynamic parameters appear similar in necrosis and pseudoprogression. However, the benefit of FET-PET lies in identifying patients requiring immediate therapeutic intervention due to progression, rather than in distinguishing between begin post-treatment changes – such as pseudoprogression and radionecrosis - that can be monitored. Some studies did not clearly differentiate between necrosis and pseudoprogression, focusing instead on distinguishing recurrence from general treatment-related changes. A retrospective study by Lohmeier et al. involving 42 patients with low- and high-grade gliomas found that a TBRmax threshold of 2 provided a sensitivity of 81% and specificity of 60% in differentiating glioma recurrence from post-treatment changes (60). In a retrospective study by Puranik et al., TBR_max_ cutoff value of 2.5 in patients with grade 3 and 4 gliomas yielded a sensitivity of 91.6% and a specificity of 76.9% in distinguishing tumor recurrence from post-treatment changes (61). Verger et al., in a study of 31 low- and high-grade glioma patients reported that a TBR_max_ of 2.61 yielded a sensitivity of 80%, and a specificity of 86% in distinguishing progression from treatment-related changes (62).

These varying results may stem from methological differences between centers, including how background activity is defined (63). A retrospective study comparing five methods for differentiating pseudoprogression from true progression more than 12 weeks post-radiotherapy found similar diagnostic performance across approaches, with AUCs ranging from 0.80 to 0.88 (64). Another study demonstrated variability in background activity measurement depending on the region-of-interest method (2D-ROI vs. VOI 3cm diameter vs. crescent-shaped VOI), with the crescent-shaped VOI yielding the most consistent results (65). Most of these studies were conducted before the establishment of the PET RANO 1.0 criteria or EANM/EANO/RANO/SNMMI guidelines, which recommended using a crescent-shaped VOI in the contralateral frontal lobe including healthy white and gray matter for background activity assessment (28, 29).

## 6 Treatment response assessment based on FET-PET after reirradiation

Most patients with glioblastoma experience tumor recurrence shortly after initial treatment. However, there is scarce evidence regarding the use of FET-PET in the context of reirradiation.

A systematic review demonstrated that amino acid PET, including FET-PET, has superior prognostic value compared to MRI using RANO criteria in predicting OS in patients treated with bevacizumab for recurrent glioma. FET-PET predicted 9-month OS with a sensitivity of 76% (95% CI 60–87) and specificity of 71% (95% CI 53–83), whereas MRI yielded a sensitivity of 32% (95% CI 19–48) and specificity of 82% (95% CI 66–92) (66).

Several studies have showed that changes in static and dynamic FET-PET parameters can predict prognosis. However, the results are inconsistent. This variability is likely due to small patient populations and retrospective nature of most studies.

In a retrospective study by Fleischmann et al., 72 patients with recurrent high-grade glioma underwent FET-PET prior to re-irradiation with or without bevacizumab (at least 6 months after the initial radiotherapy). A shorter TTP_min_ prior to treatment was associated with shorter post-recurrence survival (PRS): 6 months for TTP_min_ <12,5min, 7 months for TTP_min_ 12.5–25 min and 11 months for TTP_min_ >25 min (p=0.027). Early TBR_max_ was not predictive of PRS (67).

Another retrospective study by Niyazi et al. included 56 patients with recurrent malignant glioma who underwent FET-PET before re-irradiation with or without bevacizumab. The study reported a significant decrease in median TBR_max_ (3.3 vs 2.6, p <0.001) and BTV (13.7 cc vs 7.3 cc, p = 0.006) after re-irradiation, but without significant impact on PFS or survival. TBR_mean_ values also did not correlate with survival. However, patients with pretherapeutic decreasing FET kinetics had worse survival compared to those with other kinetics (p = 0.01) (68).

A phase I clinical trial evaluated the prognostic value of FET-PET in 31 patients undergoing re-irradiation for high grade glioma. Baseline BTV and MRI volume were both prognostic for OS (HR = 1.3 p < 0.01 and HR = 1.3 p < 0.01, respectively). However, changes in BTV and TBR_max_ were not correlated with survival (69).

In a prospective study by Galldiks et al., 21 glioblastoma patients with recurrent glioblastoma undergoing bevacizumad with lomustine therapy (without re-irradiation) were monitored using FET-PET. A reduction in TBRmax > 27% and TBR _mean_ > 17% predicted improved OS > 9 months (sensitivity 92%, specificity 63% for both; p = 0.036 and p=0.02, respectively) Additionally, an absolute MTV below 5 ml at follow-up was associated with improved OS (12 vs. 6 months, sensitivity 85%; specificity, 88%; p < 0.001). MRI-based response assessment did not predict OS (7).

The role of FET-PET in the assessing response to re-irradiation remains limited due to the lack of significant correlation reported in current studies.

In a phase I trial, Moller et al. used FET-PET to monitor results of stereotactic radiotherapy for recurrent glioblastoma. While pre-treatment TBR_max_ and BTV were associated with better outcomes, changes in this parameter did not predict survival. Similar findings were reported by Niyazi in patients undergoing re-irradiation with bevacizumab. These findings indicate the need for further studies. Recent data suggest that early-phase FET-PET imaging may reflect the most aggressive tumor regions (70, 71). High uptake in early acquisition is more commonly observed in IDH-wildtype gliomas, and longer TTP appears to have a favorable prognostic impact (72). A retrospective study in patients treated with TTFields (tumor treating fields) in recurrent tumors demonstrated the value FET-PET monitoring. Increased uptake was observed in time of further progression compared to baseline (TBR_max_ 3.5 ± 0.6, range 2.5–4.4; TBR_mean_ 2.7 ± 0.7, range 2.0–4.0). In patients treated with TTFields as a maintenance therapy without progression, FET-PET showed either reduced metabolic activity or no increased uptake at follow-up (73).

Abovementioned studies have been summarized in **Table 2**.

**Table 2:** Overview of studies analyzing FET-PET after reirradiation.

Study	N of pts	Time of PET after last therapy	Evaluated parameters	Dynamic vs static acquisition	Prognostic of OS or PFS
Sogani et al. (42)	32	ND	TBR_max_,TBR_mean_,	Static	Not assessed
Pyka et al. (43)	47		TBR_mean_, TTP	Static and dynamic	Not assessed
Vidmar et al. (44)	47	Min. 12 weeks	TBR_max_,TBR_mean_, TTP	Static and dynamic	Not assessed
Bashir et al. (45)	146	6 months	TBR_max_, TBR_mean_, BTV	Static	Increasing BTV associated with shorter OS. PET parameters higher in recurrence than in posttreatment changes.
Werner et al. (46)	48	16 weeks	TBR_max_, TBR_mean_, TTP	Static and dynamic	TBRs <1.95 at suspected progression predicted longer survival.
Celli et al. (47)	45	12 weeks	TBR_max_, MTV, TTM, TTP, TAC	Static and dynamic	No impact of FET-PET parameters on OS/PFS.
Maurer et al. (48)	127	103 days	TBR_max_, TBR_mean_, TTP, slope	Static and dynamic	Longer OS in treatment related changes.
Galdiks et al. (56)	22	12 weeks	TBR_max_, TBR_mean_, TTP, TAC	Static and dynamic	TBR_max_ <2.3 predicted longer OS
Kebir et al. (57)	26	3 months	TBR_max_, TBR_mean_, TTP, TAC	Static and dynamic	Not assessed
Werner et al. (58)	23	12 weeks	TBR_max_, TBR_mean_, TTP, slope	Static and dynamic	Not assessed
Mihovilovic et al. (59)	36	12 weeks	TBR_mean_, TBR_max_	Static	Not assessed
Lohmeier et al. (60)	42	ND	TBR_mean_, TBR_max_	Static	Not assessed
Puranik et al. (61)	171	min. 3 months	TBR_max_	Static	Not assessed
Verger et al. (62)	32	16 months	TBR_max_, TBR_mean_, TTP, slope	Static and dynamic	Not assessed
Fleischmann et al. (67)	72	6 months	TBR_max_, BTV, TAC, TTP	Static and dynamic	Longer TTP before reirradiation connected with longer post-recurrence survival.
Niyazi et al. (68)	56	6 months	SUV_max_/BG, SUV_mean_/BG, TAC	Static and dynamic	Increasing TAC prior to re-irradiation correlated with longer survival.
Moller et al. (69)	31	6 months	BTV, T_max_/B	Static	Baseline BTV prognostic for OS.
Galldiks et al. (7)	21	9–11 days before bevacizumab/lomustine inintiation and after 8–10 weeks	TBR_mean_, TBR_max_, MTV	Static	TBR_max_, TBR_mean_ and MTV reduction correlated with longer OS.
Ceccon et al. (73)	12	ND	TBR_max_, TBR _mean_	Static	Not assessed

TBR, tumor to background ratio; B/BG, background; BTV, biological tumor volume; TAC, time activity curve; TTP, time to peak; TTM, total tumor metabolism; MTV, metabolic tumor volume; OS, overall survival; PFS, progression free survival.

## 7 Conclusions

Growing evidence supports the efficacy of FET-PET in accurate selection for reirradiation. Based on current data, FET-PET can reliably distinguish post-treatment changes from true tumor progression and may enhance target definition by identifying areas of tumor infiltration beyond contrast enhancement on MRI. Most studies suggest that TBR_mean_ and TBR_max_ above 2.0 should be considered indicative of progression or metabolically active disease following primary radiotherapy. In the PET RANO classification, stable disease corresponds to stable uptake after treatment. However, it may still reflect a metabolically active tumor. Careful evaluation is crucial, and optimal management for this subgroup remains unclear. Future research should explore whether additional therapies for metabolically stable glioblastoma could lead to improved outcomes. The utility of FET PET in monitoring patients after re-irradiation also warrants further investigation.
